# Overexpression of *OsERF106MZ* promotes parental root growth in rice seedlings by relieving the ABA-mediated inhibition of root growth under salinity stress conditions

**DOI:** 10.1186/s12870-023-04136-8

**Published:** 2023-03-16

**Authors:** Hung-Chi Chen, Shi-Cheng Huang, Yen-Fu Chen, Che-Wei Kuo, Ying-Hsuan Chen, Men-Chi Chang

**Affiliations:** Department of Agronomy, National Taiwan University, Taipei, Taiwan, ROC

**Keywords:** ABA, AP2, ERF, Root growth, Salinity stress, Transcription factor

## Abstract

**Background:**

Roots are essential for plant growth and have a variety of functions, such as anchoring the plant to the ground, absorbing water and nutrients from the soil, and sensing abiotic stresses, among others. *OsERF106MZ* is a salinity-induced gene that is expressed in germinating seeds and rice seedling roots. However, the roles of OsERF106MZ in root growth remain poorly understood.

**Results:**

Histochemical staining to examine β-glucuronidase (GUS) activity in transgenic rice seedlings harboring *OsERF106MZp::GUS* indicated that *OsERF106MZ* is mainly expressed in the root exodermis, sclerenchyma layer, and vascular system. *OsERF106MZ* overexpression in rice seedlings leads to an increase in primary root (PR) length. The phytohormone abscisic acid (ABA) is thought to act as a hidden architect of root system structure. The expression of the ABA biosynthetic gene *OsAO3* is downregulated in *OsERF106MZ*-overexpressing roots under normal conditions, while the expression of *OsNPC3*, an *AtNPC4* homolog involved in ABA sensitivity, is reduced in *OsERF106MZ*-overexpressing roots under both normal and NaCl-treated conditions. Under normal conditions, *OsERF106MZ*-overexpressing roots show a significantly reduced ABA level; moreover, exogenous application of 1.0 µM ABA can suppress OsERF106MZ-mediated root growth promotion. Additionally, *OsERF106MZ*-overexpressing roots display less sensitivity to ABA-mediated root growth inhibition when treated with 5.0 µM ABA under normal conditions or exposed to NaCl-treated conditions. Furthermore, chromatin immunoprecipitation (ChIP)-qPCR and luciferase (LUC) reporter assays showed that OsERF106MZ can bind directly to the sequence containing the GCC box in the promoter region of the *OsAO3* gene and repress the expression of *OsAO3*.

**Conclusions:**

*OsERF106MZ* may play a role in maintaining root growth for resource uptake when rice seeds germinate under salinity stress by alleviating ABA-mediated root growth inhibition.

**Supplementary Information:**

The online version contains supplementary material available at 10.1186/s12870-023-04136-8.

## Background

Rice (*Oryza sativa* L.) is a major cereal staple food for more than half of the world’s population. To date, rice has been grown in at least 100 countries across a diverse range of ecosystems, such as irrigated/paddy, lowland rainfed, deepwater/flood-prone, and upland systems [1]. Therefore, the developmental plasticity of the root system architecture (RSA) is critical for rice fitness under varied environmental conditions. Rice root systems play an essential role in the acquisition of water and nutrients from the external medium, which is strongly related to plant growth and grain yield. It is noteworthy that the root system of rice is more complex than those of other cereal crops. Rice has a fibrous root system consisting of an ephemeral seminal root (SR), nodal roots (NRs), and lateral roots (LRs). The SR originates from the embryo and survives for only approximately one month after germination. NRs, also known as adventitious or crown roots, typically emerge from the lowermost internode of the stem. Both SR and NRs produce LRs. LRs can be further classified into two distinct types: thick LRs (TLRs, also known as L-type LRs) and fine LRs (FLRs, also known as S-type LRs) [2, 3]. TLRs may branch and have the ability to form new LRs, while FLRs do not have the ability to create branches. Furthermore, the modulation of RSA is coordinately determined by genetic, phytohormonal, metabolic, and environmental cues [4]. For example, a T-DNA insertion mutant of *AtNPC4* (*nonspecific phospholipase C4*, At3g03530) has a sensitive defect in the abscisic acid (ABA)-mediated inhibition of root growth and shows a decrease in tolerance to high salinity and water deficiency [5]. The knockout of the *AUXIN RESPONSE FACTOR12* gene (*OsARF12*, Os04g0671900) reduces the expression of auxin biosynthetic genes (*OsYUCCAs*) and auxin efflux carriers (*OsPINs* and *OsPGPs*), resulting in a decrease in auxin levels and primary root (PR) length [6]. A loss-of-function mutant of *OsWRKY28* (Os06g0649000), a transcription factor (TF), exhibits decreases in the LR number and total root length, which may be caused by an effect on jasmonic acid (JA) homeostasis [7]. In addition, notably, compared with stress-sensitive rice cultivars, stress-tolerant rice cultivars could promote more LR development in response to transient waterlogged-drought conditions [8]. This plastic root trait of stress-tolerant rice cultivars is beneficial to dry matter production through enhancement of water uptake under osmotic stress conditions. However, the molecular mechanism by which RSA is modulated remains elusive.

ABA is a phytohormone that regulates many plant physiological and developmental processes, such as seed dormancy, stomatal closure, leaf senescence, and stress tolerance [9–11]. Moreover, ABA is described as the “hidden architect of root system structure” [12]. ABA shapes the structure of root system by regulating the production of LRs and controlling root elongation. Almost all the enzymes involved in the main ABA biosynthetic pathway have been identified [13]. The first committed step of ABA biosynthesis is the oxidative cleavage of a C_40_ 9-*cis*-epoxycarotenoid into a C_15_ xanthoxin (a precursor of ABA) and a C_25_ byproduct, which is carried out in plastids by 9-*cis*-epoxycarotenoid dioxygenase (NCED) [14]. Subsequently, xanthoxin is converted to abscisic aldehyde (an intermediate) by short-chain alcohol dehydrogenase/reductase (SDR), which is encoded by the *ABSCISIC ACID DEFICIENT2* (*ABA2*) gene, in the cytosol [15, 16]. Finally, abscisic aldehyde oxidase (AAO) is responsible for ABA production [17]. Indeed, several studies have shown that the molecular manipulation of ABA biosynthetic genes can not only affect endogenous ABA levels and stress responses in transgenic plants but also alter their root architecture. For example, the ectopic expression of *Brassica napus NCED3* in *Arabidopsis* elevates cellular ABA levels and reduces LR initiation [18]. The clustered regularly interspaced short palindromic repeat (CRISPR)/CRISPR-associated protein 9 (Cas9)-mediated mutation of *OsNCED3* could promote seed germination, increase root growth, and decrease tolerance to salinity and osmotic stresses in transgenic rice [19]. The overexpression of *OsAO3* (*aldehyde oxidase 3*), an *AtAAO3* (At2g27150) homolog, increases cellular ABA levels and reduces root growth in transgenic rice, while a loss-of-function mutation of *OsAO3* decreases cellular ABA levels and stimulates root growth [20]. However, little is known about the transcriptional regulation of ABA biosynthetic genes in relation to the root architecture of plants.

APETALA2/ethylene-responsive factor (AP2/ERF) proteins constitute one of the largest TF families in plants. To date, at least 170 AP2/ERFs have been identified in both *Arabidopsis* and rice. AP2/ERF proteins are characterized by the presence of at least one copy of the AP2/EREBP (APETALA2/ethylene-responsive element binding protein) domain, a domain with a length of approximately 60 amino acids known to be involved in AP2/ERF-DNA interaction [21]. Based on the number of AP2/EREBP domains and the occurrence of other domains, AP2/ERFs can be classified into four subfamilies: the AP2, RAV (related to ABA insensitive3/viviparous1), CBF/DREB (C-repeat binding factor/dehydration-responsive element binding protein), and ERF subfamilies [22, 23]. Among these TFs, CBF/DREB members regulate the transcriptional expression of target genes by targeting the CRT/DRE (C-repeat/dehydration-responsive element [5’-TACCGACAT-3’]) box, a *cis*-regulatory element (CRE), present in their promoters, while ERF members modulate the transcriptional expression of target genes by binding to the GCC (5’-AGCCGCC-3’) box [21, 24]. Additionally, members of the CBF/DREB and ERF subfamilies may act as key regulatory hubs, integrating developmental, hormonal, and environmental signaling in the balance of plant growth and adaptation to stress [25, 26]. For example, JERF1 (Solyc06g063070) transcriptionally activates the expression of *NtSDR*, causing an increase in ABA levels, which ultimately enhances tolerance to salinity and cold stresses in tobacco [27, 28]. OsERF71 (Os06g0194000) directly activates the transcriptional expression of *OsCINNAMOYL-COENZYME A REDUCTASE1*, a lignin biosynthetic gene involved in cell wall modification to enable root morphological adaptations, which confers tolerance to drought stress in rice [29]. Recently, a total of six AP2 members and six ERF members in green algae (*Auxenochlorella protothecoides*) have been suggested to be involved in the crosstalk between temperature stress and lipid metabolism [30]. However, the physiological and regulatory roles of most OsERFs in rice are still unclear.

In our previous study, we isolated a novel ERF-X-type TF, *OsERF106MZ*, and showed that its expression is upregulated under salinity stress conditions [31]. Moreover, OsERF106MZ might act as a negative regulator of shoot growth and the salinity stress response in rice. Notably, *OsERF106MZ* is predominantly expressed in the roots of rice seedlings, but its roles in regulating root growth and development remain unclear. In the present study, our results indicate that OsERF106MZ promotes the root growth of young rice seedlings by relieving the ABA-mediated inhibition of root growth under salinity stress conditions.

## Results

### Overexpression of OsERF106MZ promotes root growth in transgenic rice seedlings

**Fig. 1:**
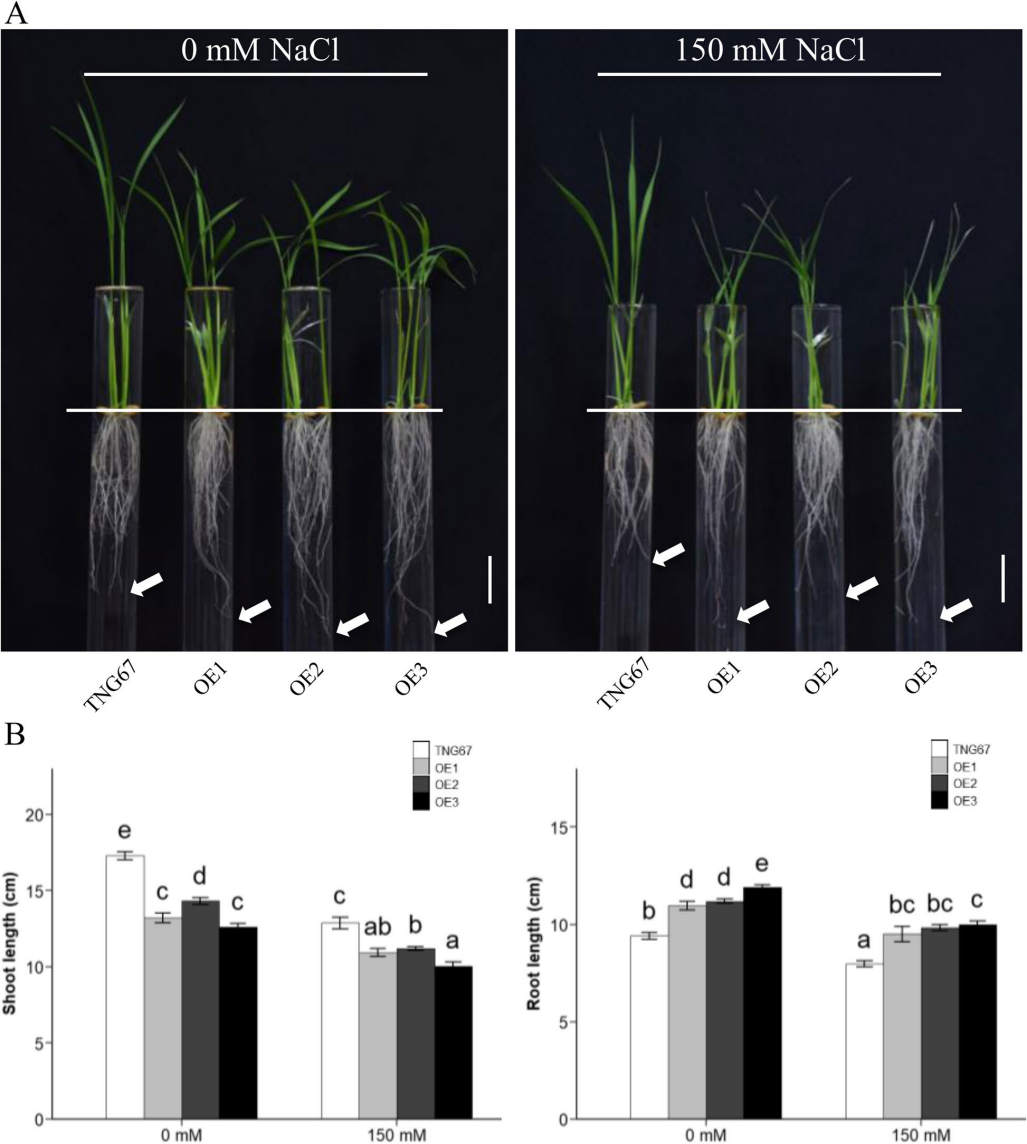
A comparison of growth patterns between *OsERF106MZ*-overexpressing transformants (OE1 to OE3) and the corresponding WT (TNG67) under normal (0 mM NaCl) and NaCl-treated (150 mM NaCl) conditions. **a** Phenotypic analysis of OE lines and TNG67 at the seedling stage. **b** Quantification of shoot length (left panel) and seminal root length (right panel) in seedlings of the OE lines and TNG67 grown under normal and NaCl-treated conditions. The seedlings were grown on basal medium for 7 days and then transferred to basal medium containing 0 or 150 mM NaCl for an additional 2 days. The shoot and root length values are the mean ± SE of 3 independent biological replicates. Each biological replicate contained 15 seedlings. The basal medium used in this experiment was half-strength Kimura B solution

**Fig. 2:**
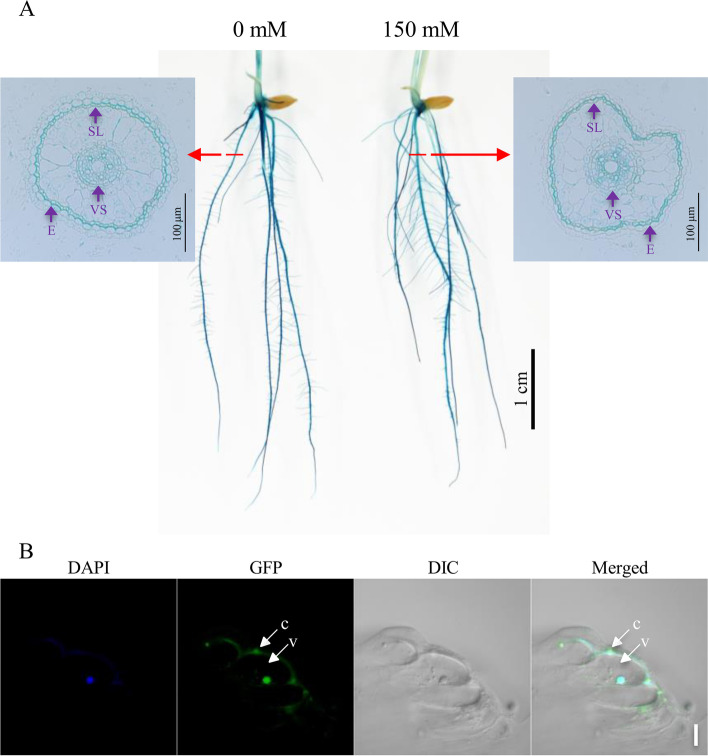
The spatial expression of *OsERF106MZ* and the subcellular localization of OsERF106MZ-GFP in stable rice transformants. **a** The spatial expression of *OsERF106MZ* in the roots of rice seedlings grown under normal and NaCl-treated conditions. E, exodermis; SL, sclerenchyma layer; VS, vascular system. Seven-day-old seedlings harboring *OsERF106MZp::GUS* were treated with or without 150 mM NaCl for 24 hours and then subjected to histochemical staining for GUS activity. The cross-section images were taken from the maturation zone of NRs. **b** Subcellular localization of OsERF106MZ-GFP in stable transgenic calli. C, cytosol; V, vacuole. Scale bar, 20 μm. The calli induced from the husk-removed seeds harboring *35Sp::OsERF106MZ-GFP* were subjected to GFP visualization by confocal microscopy.

To explore the roles of OsERF106MZ in root growth and development, we examined the differences in PR length between Tainung 67 (TNG67 [WT]) and three independent *OsERF106MZ*-overexpressing transgenic rice lines (OE1 to OE3) at the young seedling stage. First, the relative mRNA levels of *OsERF106MZ* were found to be significantly higher in transgenic rice seedlings harboring either *35Sp::OsERF106MZ* (OE1) or *35Sp::OsERF106MZ-green fluorescent protein* (*GFP*) (OE2 and OE3) than in TNG67 seedlings (Additional file 1: Figure S1a) under both normal and NaCl-treated conditions. Moreover, Western blotting using an anti-GFP antibody revealed that OsERF106MZ-GFP fusion proteins were not detectable in either TNG67 or OE1 seedlings but were obviously expressed in both OE2 and OE3 seedlings (Additional file 1: Figure S1b). Further phenotypic analysis showed that the shoot length of the *OsERF106MZ*-overexpressing transgenic rice seedlings was significantly shorter than that of the TNG67 seedlings, which was consistent with our findings reported in a previous study [31] (Fig. 1a and b). Under normal (0 mM NaCl) conditions, the PR lengths of three independent *OsERF106MZ*-overexpressing transgenic rice lines were approximately 11.0 cm, 11.2 cm, and 11.9 cm, while the PR length of TNG67 seedlings was only approximately 9.4 cm (Fig. 1a and b). Although the PR lengths of both TNG67 seedlings and *OsERF106MZ*-overexpressing transgenic rice seedlings decreased under NaCl-treated (150 mM NaCl) conditions, the PR lengths of three independent NaCl-treated *OsERF106MZ*-overexpressing transgenic rice lines were still significantly longer than those of the NaCl-treated TNG seedlings (Fig. 1b). These data revealed that OsERF106MZ plays a positive role in promoting the root growth of rice seedlings. Incidentally, histochemical staining of β-glucuronidase (GUS) activity in transgenic rice seedlings harboring *OsERF106MZp::GUS* indicated that *OsERF106MZ* was predominantly expressed in the exodermis, sclerenchyma layer, and vascular system of roots; moreover, the spatial expression of *OsERF106MZ* was not significantly different between the roots of non-NaCl- and NaCl-treated transgenic seedlings (Fig. 2a). In addition, the OsERF106MZ-GFP fusion proteins not only colocalized with the nuclear stain 4’,6-diamidino-2-phenylindole (DAPI) but also were present in the cytosol of callus cells harboring *35Sp::OsERF106MZ-*GFP (Fig. 2b).

### Transcriptomic, physiological, and biochemical analyses of OsERF106MZ-overexpressing transgenic rice roots

**Fig. 3:**
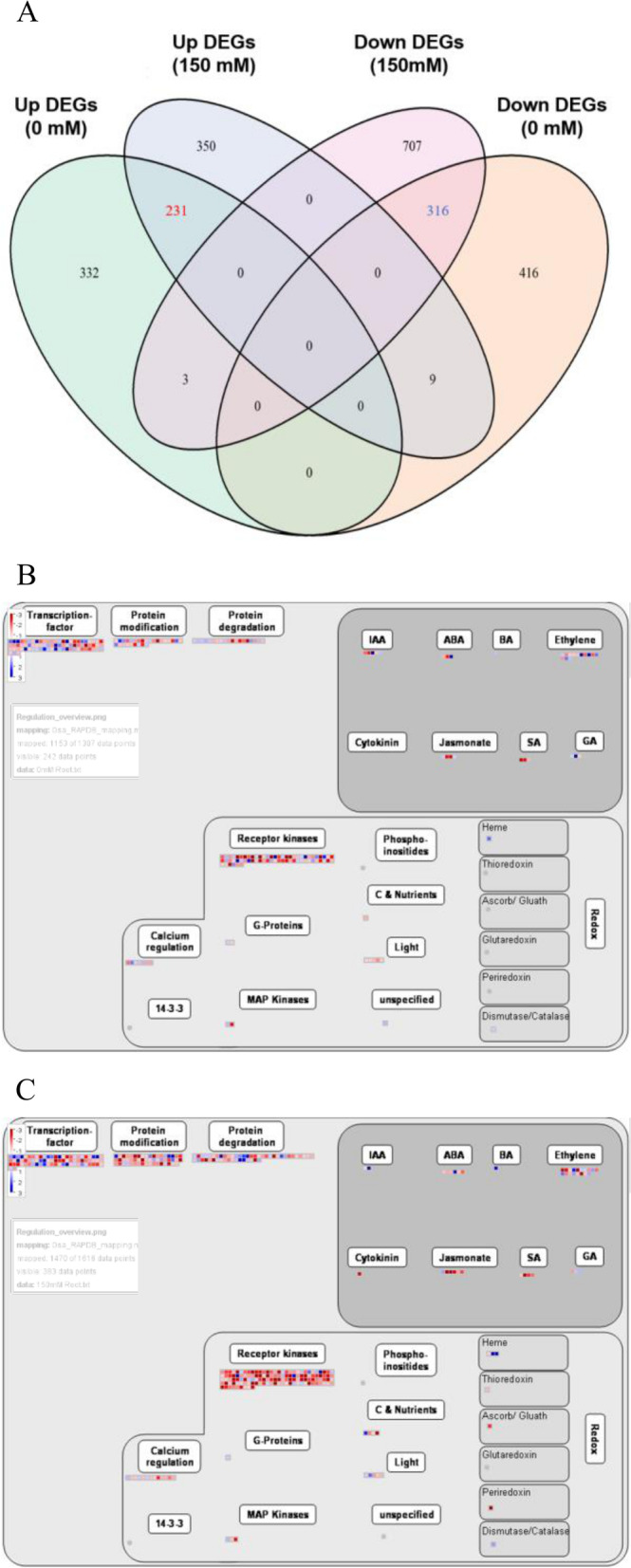
Venn diagram (**a**) and MapMan (**b**) analyses of DEGs between the roots of *OsERF106MZ*-overexpressing transformants (OE1) and TNG67. **b** MapMan overview of changes in the expression of phytohormone-related DEGs in *OsERF106MZ*-overexpressing roots grown under normal (upper panel) and NaCl-treated (lower panel) conditions. Blue and red indicate up- and downregulated gene expression, respectively

**Fig. 4:**
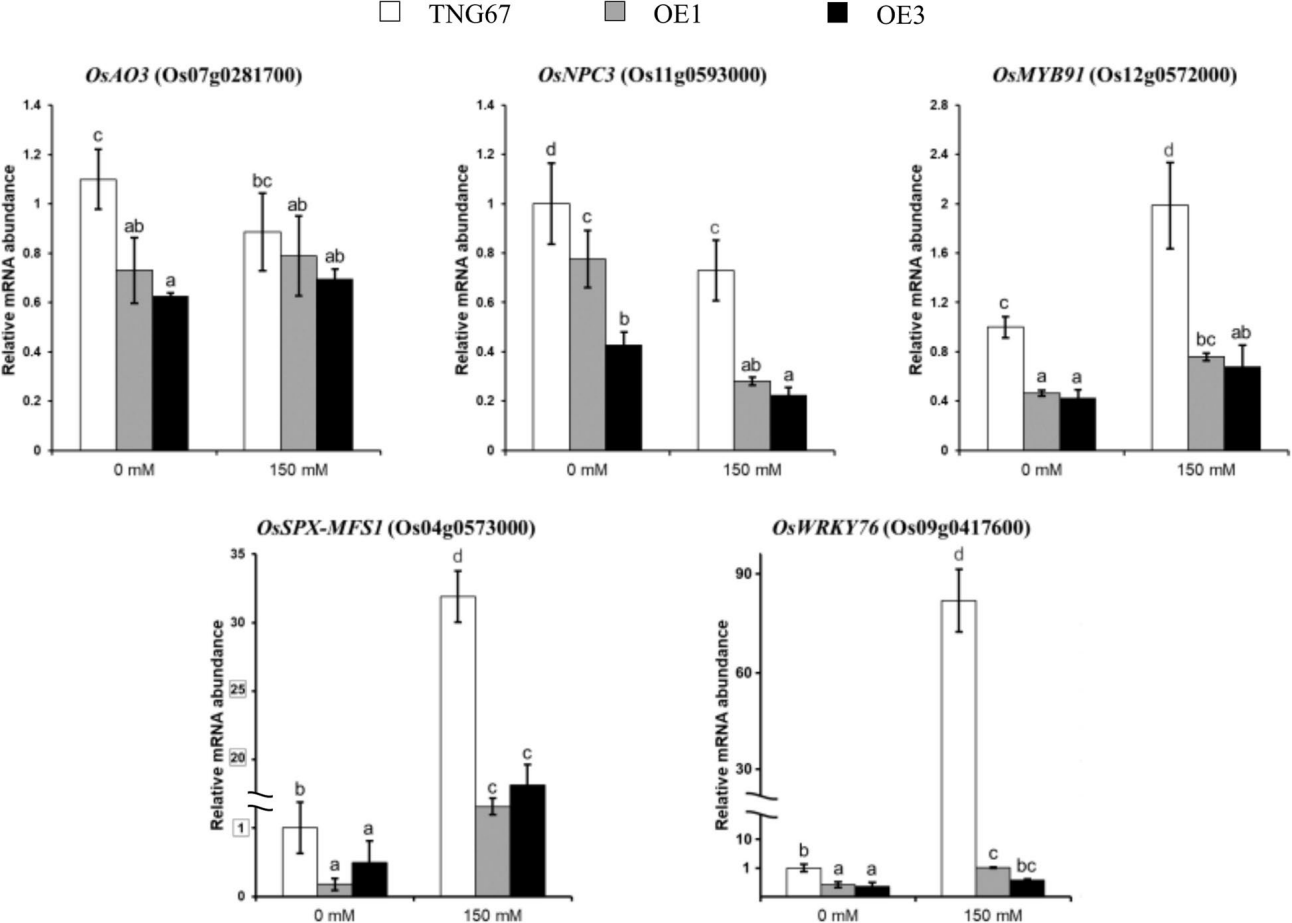
Quantification of root growth- and abiotic stress-related DEG mRNAs in the roots of two *OsERF106MZ*-overexpressing transgenic rice lines (OE1 and OE3) and TNG67 by qPCR. The values are the mean ± SE of at least five biological replicates, each with two technical replicates. The total RNA used in the qPCR and RNA-seq assays was isolated from the same roots of 7-day-old seedlings subjected to 0 or 150 mM NaCl for 1 day

**Fig. 5:**
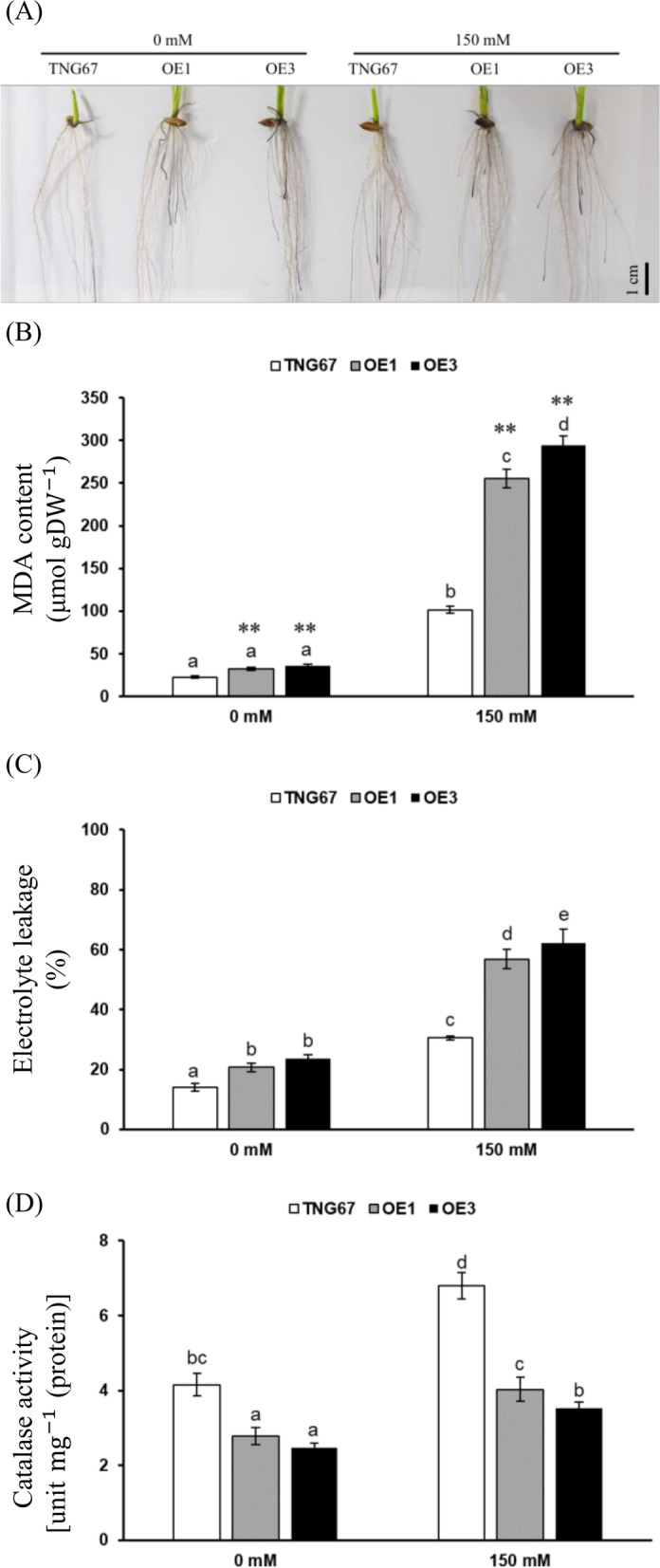
Analysis of ROS levels, MDA contents, electrolyte leakage, and CAT activity in the roots of both *OsERF106MZ*-overexpressing transgenic rice seedlings and TNG67 seedlings. **a** NBT staining. **b** MDA content. **c** Electrolyte leakage. **d** CAT activity. These physiological assays were carried out as previously described [31]. The values are the mean ± SE of five biological replicates, each with two technical replicates. The asterisks indicate significant differences (*, *P* < 0.05 and **, *P* < 0.01) in comparison to the corresponding WT based on Student’s *t* test. For these assays, seedlings were grown on basal medium for 7 days and then transferred to basal medium containing 0 or 150 mM NaCl for an additional day

To understand the mechanism of root growth promotion by OsERF106MZ, we performed a comparative transcriptomic analysis between TNG67 roots and *OsERF106MZ*-overexpressing roots using high-throughput RNA sequencing (RNA-seq). After background correction and normalization, a total of 1307 and 1616 differentially expressed genes (DEGs) were identified from the roots of *OsERF106MZ*-overexpressing transgenic rice seedlings grown under normal and NaCl-treated conditions, respectively (Fig. 3a, Additional file 2: Dataset File S1). Among these genes, 566 and 590 DEGs were upregulated in the roots of *OsERF106MZ*-overexpressing transgenic rice seedlings grown under normal and NaCl-treated conditions, respectively, while 741 and 1026 DEGs were downregulated (Fig. 3a, Additional file 2: Dataset File S1). MapMan analysis of these DEGs suggested that 31 and 41 DEGs were involved in phytohormone (e.g., auxin, ABA, ethylene [ET], gibberellins, JA, and salicylic acid) homeostasis when the *OsERF106MZ*-overexpressing transgenic rice roots were grown under normal and NaCl-treated conditions, respectively (Fig. 3b and c). Notably, only one of these phytohormone-related DEGs, *OsAO3* (Os07g0281700, an *AtAAO3* homolog), was nearly identical to its *Arabidopsis* homolog (Additional file 3: Dataset File S2). Additionally, a four-way Venn diagram showed that a total of 547 DEGs were common to the roots of *OsERF106MZ*-overexpressing transgenic rice seedlings grown under both conditions, among which 231 and 316 DEGs were consistently up- and downregulated, respectively, under both conditions (Fig. 3a, Additional file 4: Dataset File S3). Gene Ontology analysis revealed that the major ontological categories of the commonly regulated DEGs were ‘terpenoid metabolic process’ under the ‘biological process’ category, ‘mitochondrion’ under the ‘cellular component’ category, and ‘receptor activity’ under the ‘molecular function’ category (Additional file 1: Figure S2). Indeed, several genes involved in root growth and stress responses were found among these commonly regulated DEGs, such as *OsMYB91* (*myeloblastosis91* [Os12g0572000]), *OsNPC3* (a close homolog of *AtNPC4* involved in ABA sensitivity and salinity tolerance [Os11g0593000]), *OsSPX-MFS1* (*SYG1/PHO81/XPR1-Major Facility Superfamily1* [Os04g0573000]), and *OsWRKY76* (Os09g0417600). A quantitative PCR (qPCR) assay was performed to verify the accuracy of the RNA-seq data. As shown in Fig. 4, the mRNA levels of *OsAO3* were lower in *OsERF106MZ*-overexpressing roots than in TNG67 roots under normal conditions, but there were no obvious differences in the mRNA levels of *OsAO3* between TNG67 roots and *OsERF106MZ*-overexpressing roots under NaCl-treated conditions. Furthermore, the mRNA levels of *OsMYB91*, *OsNPC3*, *OsSPX-MFS1*, and *OsWRKY76* were lower in *OsERF106MZ*-overexpressing roots than in TNG67 roots under both conditions. These qPCR results were roughly consistent with the RNA-seq data. Additionally, the results of physiological and biochemical analyses revealed that superoxide anion staining with nitro blue tetrazolium (NBT), relative conductivity, and malondialdehyde (MDA) contents in the roots of *OsERF106MZ*-overexpressing transformants were significantly greater than in the same tissues of TNG67 seedlings, while the activity of catalase (CAT) in *OsERF106MZ*-overexpressing roots was lower than that in TNG67 roots (Fig. 5). Promoter analysis further indicated that *OsAO3* and 57 commonly regulated DEGs contained at least one GCC box, an ERF-binding CRE, in their 1-kb promoter regions, including *OsNPC3*, a commonly downregulated DEG (Additional file 1: Table S1, Additional file 4: Dataset File S3).

### OsERF106MZ promotes root growth by relieving the ABA-mediated inhibition of root growth

**Fig. 6:**
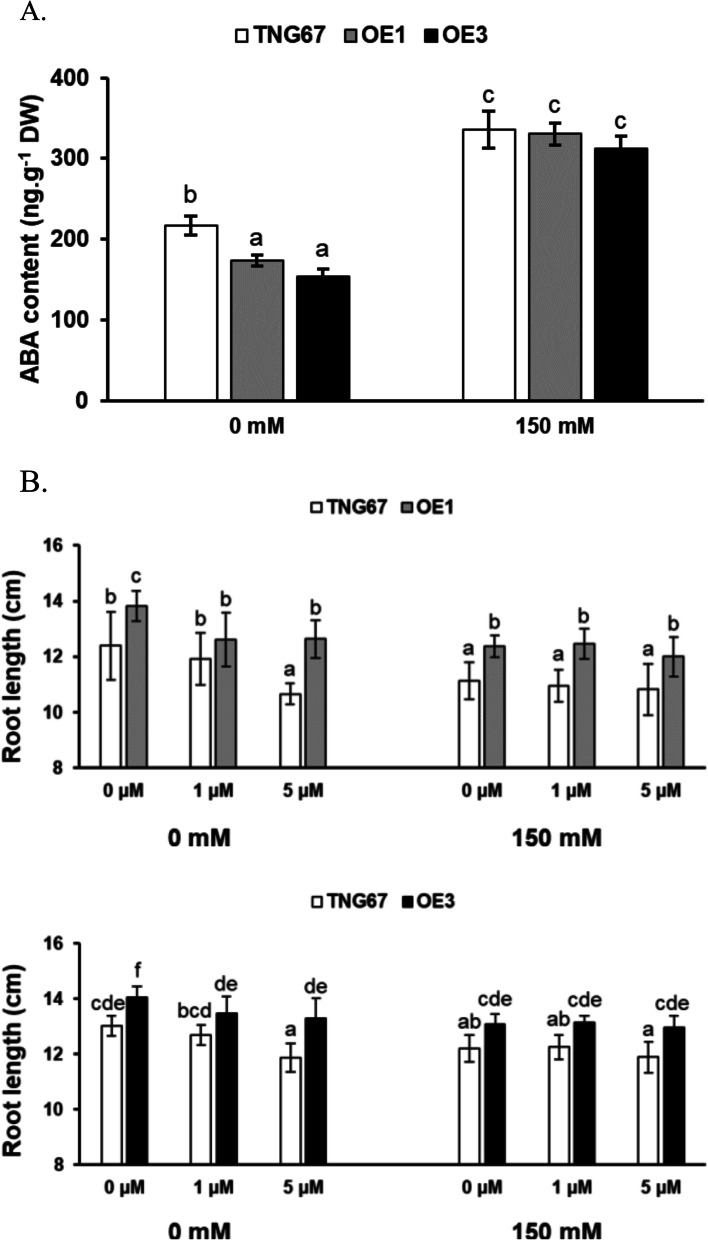
Examination of endogenous ABA contents and the response to exogenous ABA in the roots of *OsERF106MZ*-overexpressing transformants and TNG67 under normal and NaCl-treated conditions. **a** Endogenous ABA contents in the roots of both *OsERF106MZ*-overexpressing transgenic rice seedlings and TNG67 seedlings. The values are the mean ± SE of five biological replicates, each with two technical replicates. **b** Changes in the root length of *OsERF106MZ*-overexpressing transformants and TNG67 upon treatment with or without ABA. Upper panel, TNG67 vs. OE1; Lower panel, TNG67 vs. OE3. All root length values are the mean ± SE of 20 independent biological replicates (*n* = 20). The seedlings were grown on basal medium for 7 days and then transferred to basal medium containing 0 or 150 mM NaCl for an additional 2 days

Because the expression of *OsAO3* (an ABA biosynthetic gene) and *OsNPC3* (a close homolog of *AtNPC4* involved in the ABA-mediated inhibition of root growth) is downregulated in *OsERF106MZ*-overexpressing roots either under normal conditions or under both normal and NaCl-treated conditions, respectively, we therefore sought to investigate the roles of ABA in OsERF106MZ-mediated root growth promotion. First, we performed an immunological assay to examine the differences in endogenous ABA content between TNG67 roots and *OsERF106MZ*-overexpressing roots. Under normal conditions, the ABA levels in the roots of two independent *OsERF106MZ*-overexpressing transformants were approximately 0.80- and 0.71-fold lower, respectively, than those in the same tissues of TNG67 seedlings (Fig. 6a). Although the endogenous ABA content was significantly increased in both the TNG67 roots and *OsERF106MZ*-overexpressing roots under NaCl-treated conditions, no obvious differences in ABA levels were found between the NaCl-treated TNG67 roots and NaCl-treated *OsERF106MZ*-overexpressing roots (Fig. 6a). Accordingly, we examined the effects of exogenous ABA on the PR growth of TNG67 seedlings and *OsERF106MZ*-overexpressing transgenic rice seedlings grown under normal and NaCl-treated conditions. Under normal conditions, the average root length of TNG67 seedlings decreased slightly from 12.39 cm to 11.91 cm after treatment with ABA at 1.0 µM, while the average root length of *OsERF106MZ*-overexpressing transgenic rice seedlings (OE1) decreased significantly from 13.83 cm to 12.63 cm (upper panel, Fig. 6b). In addition, no statistically significant differences in root length were found between the TNG67- and *OsERF106MZ*-overexpressing transformants (OE1) grown under normal conditions when seedlings were treated with ABA at 1.0 µM. Compared to the average root length of TNG67 seedlings after treatment with ABA at 1.0 µM, that of TNG67 seedlings continuously decreased from 11.91 cm to 10.66 cm after treatment with ABA at 5.0 µM. However, the average root length of the *OsERF106MZ*-overexpressing transgenic rice seedlings (OE1) did not significantly decrease after treatment with increasing concentrations of ABA ranging from 1.0 µM to 5.0 µM. Additionally, although the root lengths of both TNG67 seedlings and *OsERF106MZ*-overexpressing transgenic rice seedlings (OE1) were significantly reduced under NaCl-treated conditions, the average root length of NaCl-treated *OsERF106MZ*-overexpressing transgenic rice seedlings was consistently longer than that of the NaCl-treated TNG67 seedlings, irrespective of treatment with or without exogenous ABA (upper panel, Fig. 6b). Similar changes in response to exogenous ABA were seen between the PRs of the TNG67 and OE3 seedlings grown under either normal or NaCl-treated conditions (lower panel, Fig. 6b). Thus, these data indicated that OsERF106MZ promotes root growth at least partly through the inhibition of ABA biosynthesis under normal conditions. However, OsERF106MZ-mediated root growth promotion could also be caused by a reduction in the sensitivity of rice seedlings to the ABA-mediated inhibition of root growth under normal and NaCl-treated conditions.

### OsERF106MZ directly binds to the promoter of OsAO3 and represses its activity

**Fig. 7:**
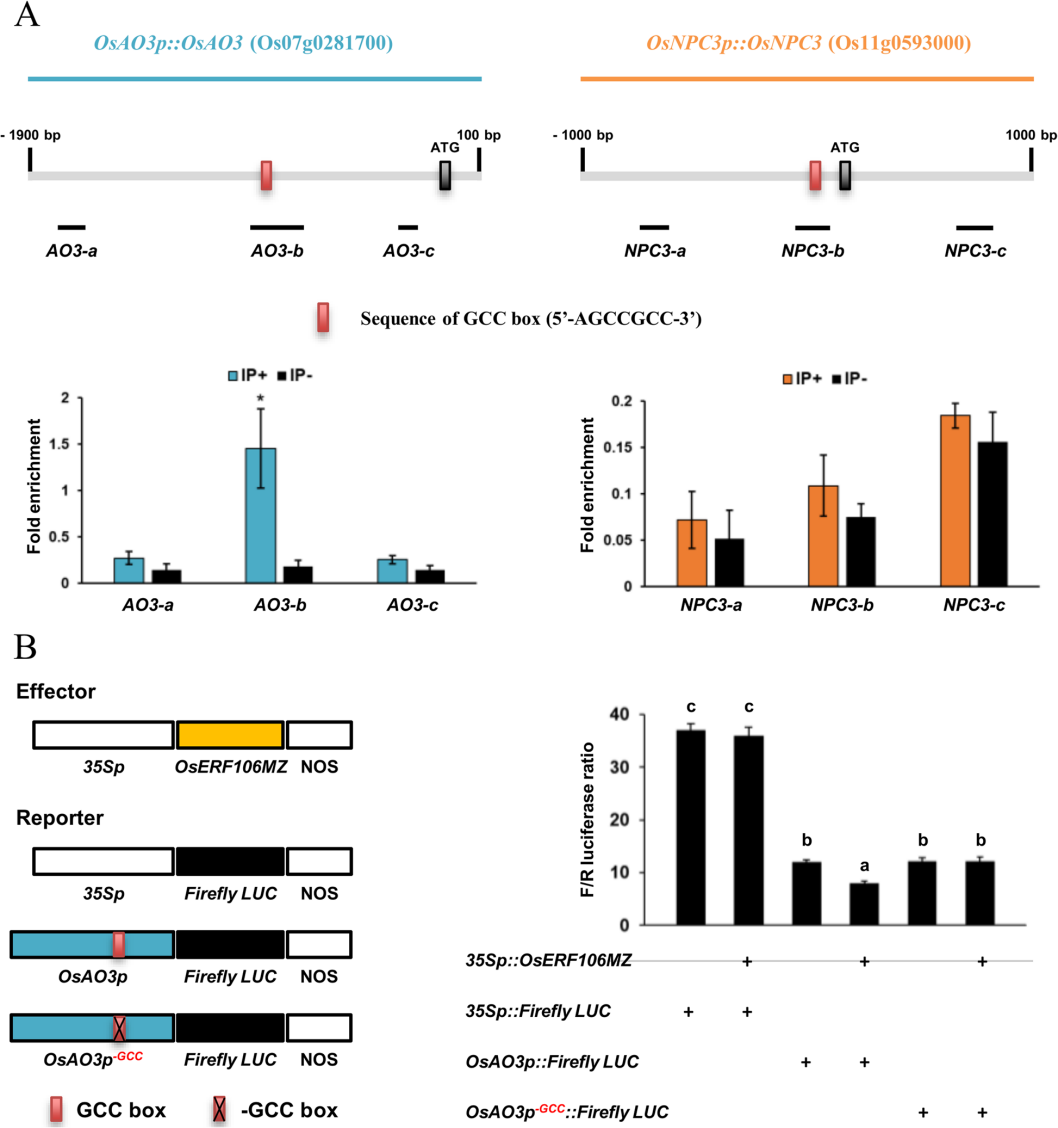
OsERF106MZ binds to the GCC box in the promoter of *OsAO3* and represses its expression. **a** Upper panel, schematic diagram of different PCR amplicons used for the ChIP-qPCR assay. Lower panel, relative fold enrichment of ChIP-qPCR data showing each PCR amplicon of *OsAO3* (left) and *OsNPC3* (right). The data are shown as the mean ± SE of at least three technical replicates. **b** Transient expression assay in rice protoplasts to examine the interaction between OsERF106MZ and the promoter of *OsAO3*. Left panel, schematic diagram of the effector and three reporter constructs used for a dual-LUC reporter assay. Right panel, transrepression of the *OsAO3* promoter by OsERF106MZ. The values are the mean ± SE (*n* = 5)

Because the *OsAO3* and *OsNPC3* genes contain a typical GCC box in their promoters (Additional file 1: Table S1, Additional file 4: Dataset File S3), we conducted a chromatin immunoprecipitation (ChIP)-qPCR assay to examine whether OsERF106MZ directly binds to the promoter regions of *OsAO3* and *OsNPC3*. As shown in Fig. 7a, OsERF106MZ specifically bound to the amplicon-b region, containing the GCC box (-657 to -651), of the *OsAO3* promoter but not to the amplicon-a and -c regions of the promoter. Additionally, there were no significant differences in the abundance of the *NPC-a*, *-b*, and *-c* amplicons between the samples immunoprecipitated with agarose beads (IP-) or with anti-GFP-conjugated agarose beads (IP +). These data suggested that OsERF106MZ can directly bind to the GCC box within the *OsAO3* promoter in vivo. To further determine whether OsERF106MZ represses the expression of *OsAO3* through binding to the GCC box of its promoter, a dual-luciferase (LUC) reporter assay was conducted. As shown in Fig. 7b, LUC activity in rice protoplasts transfected with both the *35Sp::OsERF106MZ* and *OsAO3p::Firefly LUC* plasmids was significantly lower (approximately 40% lower) than that in the protoplasts transfected with the *OsAO3p::Firefly LUC* plasmid alone. However, no obvious differences in LUC activity were observed when rice protoplasts were transfected with the *OsAO3p*^*−GCC*^*::Firefly LUC* plasmid, a modified plasmid in which the GCC box is deleted, irrespective of cotransfection with or without the *35Sp::OsERF106MZ* plasmid. These data indicated that OsERF106MZ binding to the GCC box exerts a suppressive or negative influence on *OsAO3* expression.

## Discussion

### OsERF106MZ promotes root growth at least partly by disrupting ABA biosynthesis under normal conditions

Plant roots are essential for water and nutrient uptake, anchoring and mechanical support, and storage functions, which are closely correlated with plant growth and survival. Therefore, deciphering the development and architecture of roots may be beneficial for the manipulation of root traits in improving crop productivity. The architecture of the root system is mainly determined by three factors: (1) the position of LRs, (2) the branching angle that LRs form with the parent root, and (3) root elongation. Root branching and elongation, which are responsible for the overall shape of the root system, are determined by a series of root-environment interactions throughout the life span of the plant. Interactions with the environment require sensation or perception at the root surface, conversion of extracellular cues into intracellular signals, and subsequent activation of a response within the root. To a large extent, the developmental plasticity of root systems in response to environmental changes is mediated by phytohormones [32, 33]. ABA is a ubiquitous phytohormone that is synthesized from 9-*cis*-epoxycarotenoid by a series of sequentially acting enzymes, including NCED, ABA2/SDR, and AAO. ABA is known to regulate many physiological and developmental processes in plants, such as seed germination and dormancy, stomatal movement, leaf senescence, and adaptation to stress [10, 13]. When plants encounter abiotic stresses, ABA accumulates via de novo synthesis, which in turn triggers stress responses, such as stomatal closure. These stress-induced increases in ABA levels can also modulate the root growth and architecture in response to abiotic stresses. In general, an intermediate level of ABA stimulates roots to grow deeper and seek water under drought conditions, while a high level of ABA inhibits root growth and LR formation [34]. Indeed, numerous studies have demonstrated that ABA plays dual roles in regulating root growth depending on its concentration [12, 35]: low concentrations of ABA stimulate root growth, but high concentrations inhibit root growth. Therefore, ABA seems to play a role in coordinating the adjustment of root growth in response to abiotic stresses. To date, several rice TFs have been shown to be involved in ABA-mediated physiological or developmental processes by directly regulating the transcriptional expression of *OsNCEDs*. For example, phosphorylated OsbZIP23 directly activates the expression of *OsNCED4*, which can increase cellular ABA levels and improve drought tolerance in rice [36]. OsNAC2 participates in ABA-induced leaf senescence by directly upregulating the expression of *OsNCED3* and directly downregulating the expression of *OsABA8ox1*, an ABA catabolic gene [37]. OsWRKY50 is involved in regulating ABA-mediated seed germination through direct transcriptional repression of *OsNCED5* [38]. OsERF19 directly binds to the promoter of *OsNCED5* to activate its expression, which increases ABA levels and improves tolerance to salt stress in rice [39]. Beyond these TFs that directly regulate the expression of *OsNCEDs*, the identification of which TFs directly regulate the expression of other ABA biosynthetic genes is relatively rare. TFs are proteins that bind specifically to CREs in the promoters of their target genes, which can either activate or inhibit gene expression. Typically, a TF follows a dimer pathway in binding to CRE. In plants, many TFs can both form homo and heterodimers, thus exerting dual or pleiotropic effects on plant growth or development. For example, the POSITIVE REGULATOR OF GRAIN LENGTH1 (PGL1)-ANTAGONIST OF PGL1 (APG) heterodimer, an OsHLH-OsbHLH complex, increases grain length and weight, whereas the APG homodimer decreases grain length and weight [40]. The octadecanoid-responsive Arabidopsis 59 (ORA59)-related to AP2 3 (RAP2.3) heterodimer, an AtERF#094 (At1g06160)-AtERF#072 (At3g16770) complex, is involved in the ET-induced triple response, while the ORA59-AtWRKY20 heterodimer, an AtERF-AtWRKY complex, is involved in JA-mediated defenses against herbivores [23, 41, 42]. These examples illustrate that the functions or regulatory roles of TFs depend, at least to some extent, on their interacting proteins. In the present study, we found that the overexpression of *OsERF106MZ* could promote root growth in transgenic rice seedlings (Fig. 1a and b). RNA-seq and qPCR analyses indicated that the expression of *OsAO3* was downregulated in *OsERF106MZ*-overexpressing roots compared to that in TNG67 roots under normal conditions; however, there was no significant difference in the expression of *OsAO3* between TNG67 roots and *OsERF106MZ*-overexpressing roots under NaCl-treated conditions (Figs. 3a and 4, Additional file 2: Dataset File S1, Additional file 3: Dataset File S2). OsAO3 is an AtAAO3 homolog and has been demonstrated to be involved in ABA biosynthesis and the ABA-mediated inhibition of root growth in rice [20]. Indeed, ABA levels in *OsERF106MZ*-overexpressing roots were approximately 20–30% lower than those in TNG67 roots under normal conditions (Fig. 6a). Moreover, the exogenous application of ABA at 1.0 µM was sufficient to suppress the OsERF106MZ-mediated promotion of root growth in transgenic rice seedlings under normal conditions (Fig. 6b). Further ChIP-qPCR studies and dual-LUC reporter assays showed that OsERF106MZ directly bound to the promoter of *OsAO3*, thus downregulating its expression (Fig. 7). Therefore, these data reveal that OsERF106MZ promotes root growth in rice seedlings at least partly by inhibiting ABA biosynthesis by directly downregulating the expression of *OsAO3* under normal conditions. Notably, the OsERF106MZ-mediated promotion of root growth seemed to be independent of ABA biosynthesis under NaCl-treated conditions because no obvious differences in the levels of either *OsAO3* transcripts or endogenous ABA were found between the NaCl-treated TNG67 roots and NaCl-treated *OsERF106MZ*-overexpressing roots (Figs. 4 and 6a). This implies that the expression of *OsAO3* for ABA biosynthesis is differentially regulated under normal and salinity stress conditions. Interestingly, the expression of both *GhNCED3a* and *GhNCED3c* for ABA biosynthesis also seemed to be differentially regulated under normal and drought stress conditions [43]. In cotton (*Gossypium* spp.), GhirNAC2 can bind to the promoters of *GhNCED3a/3c* to upregulate their expression. However, relative to the corresponding WT plants, the ABA content was decreased in *GhirNAC2*-knockdown transgenic plants under drought stress conditions, but not under normal conditions. Therefore, we speculate that OsERF106MZ likely interacts with different proteins when rice seedlings are exposed to varied environmental conditions, leading to the differential regulation of *OsAO3* under normal and NaCl-treated conditions.

### OsERF106MZ promotes root growth by reducing sensitivity to the ABA-mediated inhibition of root growth

In general, proteins encoded by homologous genes play similar roles in regulating plant physiological and developmental processes. For example, AtbHLH112 increases the expression of the *AtPOD* (*peroxidase*) and *AtSOD* (*superoxide dismutase*) genes to improve reactive oxygen species (ROS) scavenging ability, thus imparting *Arabidopsis* plants with salinity and drought tolerance [44]. AhbHLH112, an AtbHLH112-homologous protein in peanut (*Arachis hypogaea* L.), directly binds to and activates the promoter of the *AhPOD* gene, and *AhbHLH112*-overexpressing transgenic plants therefore also show greater tolerance to drought stress than WT plants [45]. Likewise, CBFs/DREBs confer cold stress tolerance upon various plant species, including *Arabidopsis* and rice [46]. In this study, we found that the root length of TNG67 seedlings decreased dramatically under normal conditions after treatment with increasing concentrations of ABA ranging from 1.0 µM to 5.0 µM, but the root length of *OsERF106MZ*-overexpressing transgenic rice seedlings was not significantly decreased (Fig. 6b). Additionally, although the salt-induced increases in ABA levels were comparable between TNG67 roots and *OsERF106MZ*-overexpressing roots under NaCl-treated conditions, the root length of *OsERF106MZ*-overexpressing transgenic rice seedlings was still greater than that of TNG67 seedlings (Fig. 6). These results imply that the overexpression of *OsERF106MZ* led to a decrease in the sensitivity of rice seedlings to the ABA-mediated inhibition of root growth. Notably, RNA-seq and qPCR analyses indicated that the expression of *OsNPC3* was downregulated in *OsERF106MZ*-overexpressing roots compared to that in TNG67 roots under normal and NaCl-treated conditions (Fig. 4). Lipid signaling is important for triggering plant adaptive responses because the initial result of a stress stimulus is usually the hydrolysis of membrane lipids [47]. This hydrolytic reaction is mediated by phospholipases, which catalyze the initial step of phospholipid breakdown to generate multiple lipid-derived second messengers [48, 49]. Phospholipase C (PLC) is one of the most important types of lipid-hydrolyzing enzymes in plants. Based on their affinities for different substrates, plant PLCs can be divided into two categories: phosphatidylinositol-specific PLCs (PI-PLCs) and phosphatidylcholine-PLCs (PC-PLCs). However, PC-PLC can not only hydrolyze PC but can also act upon other lipids, such as phosphatidylethanolamine (PE); therefore, PC-PLC is also referred to as nonspecific phospholipase C (NPC). Among rice *NPCs*, *OsNPC3* is highly homologous to *AtNPC3*, *AtNPC4*, and *AtNPC5* and is predominantly expressed in rice roots [47]. In fact, a previous study demonstrated that AtNPC3 and AtNPC4 participate in root development [50]. AtNPC4 is also involved in the salt stress response in roots [51]. In *Arabidopsis*, the overexpression of *AtNPC4* leads to a significant increase in ABA sensitivity and confers tolerance to hyperosmotic stress, while the knockout of *AtNPC4* reduces sensitivity to the ABA-mediated inhibition of root growth and renders mutants intolerant to salinity and drought stresses [5]. Interestingly, these phenotypes of the *AtNPC4* knockout mutants are similar to those of the *OsERF106MZ*-overexpressing transgenic rice seedlings. Therefore, we speculate that OsERF106MZ promotes root growth in rice seedlings at least partly by reducing the sensitivity of ABA in the inhibition of root growth, which is achieved by downregulating the expression of *OsNPC3*, a close homolog of *AtNPC4*. Nevertheless, OsERF106MZ most likely mediates the transcriptional repression of *OsNPC3* in an indirect manner, as ChIP-qPCR assays indicated that OsERF106MZ does not directly bind to the promoter of *OsNPC3*, although its promoter contains one GCC box (Fig. 7a).

### A putative role of OsERF106MZ in root responses to salinity stress

**Fig. 8:**
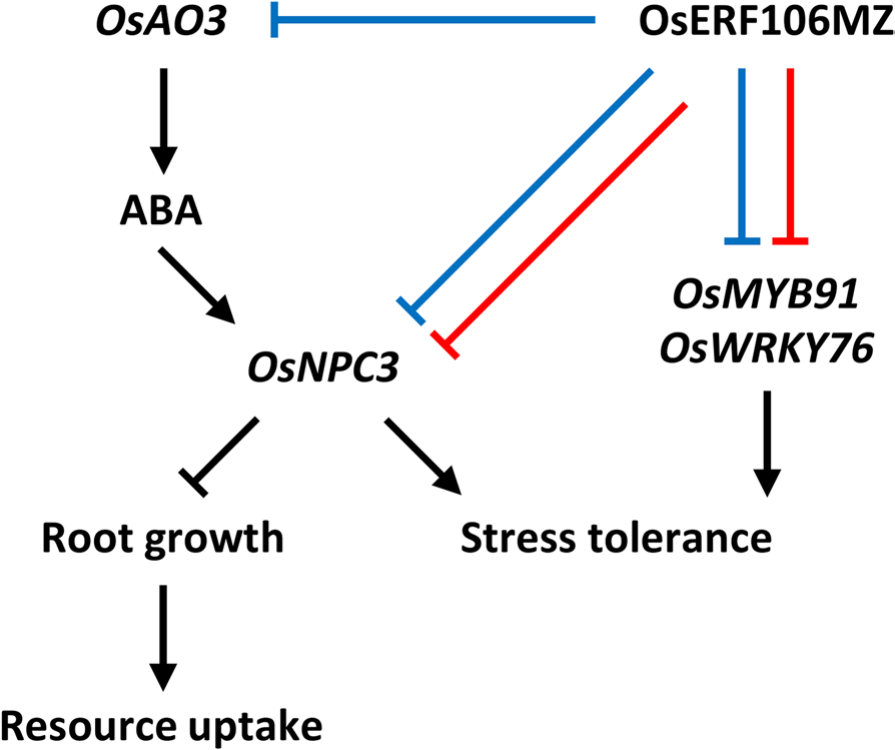
Schematic diagram summarizing the molecular mechanism underlying the OsERF106MZ-mediated regulation of stress tolerance and root growth of rice plants grown under normal (blue arrows) and NaCl-treated (red arrows) conditions

AP2/ERF TFs constitute one of the largest TF families in plants and are involved in regulating many aspects of plant growth, development, and stress responses. However, only a few ERFs, such as OsERF2 and OsERF48, have been demonstrated to be involved in the regulation of rice root architecture. OsERF2 (Os06g0181700), a subgroup Va member, is indispensable in governing root architecture and ABA-related responses by regulating the expression of genes involved in sugar metabolism and hormone signaling pathways [52]. OsERF48 (Os08g0408500), a subgroup Ib member, directly activates the expression of *OsCML16*, a calmodulin-like protein-encoding gene, which in turn increases root growth and drought tolerance [53]. Our previous study indicated that *OsERF106MZ*-overexpressing transgenic rice seedlings show reduced tolerance to salinity stress [31]. In addition to *OsNPC3*, other abiotic stress-responsive genes, such as *OsWRKY76* and *OsMYB91*, were found to be downregulated in *OsERF106MZ*-overexpressing roots (Fig. 4). A previous study showed that the overexpression of *OsWRKY76*, a group IIa WRKY gene, leads to a decrease in the resistance of rice plants to rice blast fungus (*Magnaporthe oryzae*) but improves tolerance to cold stress [54]. In addition, rice plants overexpressing *OsMYB91*, an R2R3-type MYB gene, exhibit increased tolerance to salinity stress, while *OsMYB91*-knockdown rice plants are less tolerant to salinity stress than WT plants [55]. The expression pattern of these abiotic stress-responsive genes (i.e., *OsMYB91*, *OsNPC3*, and *OsWRKY76*) in *OsERF106MZ*-overexpressing roots supports our previous finding that OsERF106MZ may act as a negative regulator of the salinity stress response by modulating the expression of stress-responsive genes. To cope with changes in the soil environment, plants need to constantly adjust their RSA to maintain efficient resource uptake. High soil salinity has a severely negative impact on root growth. When plants encounter salinity stress, both primary and lateral root growth are reduced or even stop, thus impacting resource uptake [56]. The salinity stress-induced inhibition of root growth seems to correspond to a state of quietness or inactivity known as "quiescence", which is thought to be an adaptation mechanism for coping with salinity stress [57]. In fact, the growth quiescence of roots is mediated by ABA, which depends on a series of genes required for ABA biosynthesis, signaling, and transcriptional regulation [58, 59]. In addition, even if the seeds germinate under normal conditions, they might not continue to survive without root systems. *OsERF106MZ* is a salinity-induced gene that is expressed in germinating seeds and the roots of postgermination-stage seedlings (Fig. 2; [31]). Interestingly, the overexpression of *OsERF106MZ* not only caused a reduction in the tolerance of transgenic rice seedlings to salinity stress but also alleviated the salinity stress-induced inhibition of root growth by reducing ABA biosynthesis and/or sensitivity. Therefore, the salinity-induced expression of *OsERF106MZ* seems to provide a balance between root growth and salinity tolerance. In other words, OsERF106MZ may play a role in supporting root growth for resource uptake by reducing ABA biosynthesis and/or sensitivity when rice seeds germinate under salinity stress, thus leading to a simultaneous reduction in ABA-induced salinity tolerance (Fig. 8).

## Conclusions

In the present study, we investigated the roles of a salinity-induced gene, *OsERF106MZ*, in root growth. The overexpression of *OsERF106MZ* in rice promoted parental root growth in transgenic seedlings grown under both normal and NaCl-treated conditions. In *OsERF106MZ*-overexpressing roots, the expression of *OsAO3*, an ABA biosynthetic gene, was downregulated under normal conditions, while the expression of *OsNPC3*, an *AtNPC4* homolog involved in ABA sensitivity, was reduced under both conditions. Under normal conditions, *OsERF106MZ*-overexpressing roots contained a low level of endogenous ABA; moreover, the exogenous application of ABA at 1.0 µM can suppress the OsERF106MZ-mediated promotion of root growth. However, *OsERF106MZ*-overexpressing roots were less sensitive to the ABA-mediated inhibition of root growth when they were treated with ABA at 5.0 µM. Under NaCl-treated conditions, although the salt-induced increases in ABA levels were comparable between the roots of TNG67 and *OsERF106MZ*-overexpressing transformants, the root length of *OsERF106MZ*-overexpressing transformants was consistently greater than that of TNG67 seedlings, irrespective of treatment with or without ABA. In addition, OsERF106MZ can directly bind to the GCC box in the promoter of *OsAO3* and, thus, repress its expression. Collectively, these results suggest that OsERF106MZ promotes parental root growth by alleviating the ABA-mediated inhibition of root growth.

## Materials and methods

### Plant materials, growth conditions, and root length measurements

Three independent *OsERF106MZ*-overexpressing transgenic rice lines (*35Sp::OsERF106MZ* [OE1] and *35Sp::OsERF106MZ-GFP* [OE2 and OE3]) were generated by *Agrobacterium*-mediated transformation. Among these *OsERF106MZ*-overexpressing transformants, OE1 and OE2 plants were used as the experimental materials in a previous study [31]. The genetic background of the OE plants was *Oryza sativa* L. *spp. japonica* cv. Tainung 67 (TNG67). In all the experiments, the seeds were treated with 1.5% (v/v) commercial bleach for 30 min, rinsed twice with sterile water for 30 min each time, and subsequently subjected to imbibition at 37 °C for 3 days in the dark. After imbibition, the germinated seeds were grown on a wire stand in a plastic basin with a volume of 4 L and cultured in a growth chamber (GC-1065H, FIRSTEK, Taipei, Taiwan) under long-day (16-h light/8-h dark) conditions with a day/night temperature of 28/24 °C, a relative humidity of 70%, and a light intensity of approximately 100 µE s^−1^ m^2^. After 7 days of growth, the seedlings were transferred to a glass tube with a height of 25 cm allowing their roots to grow freely inside the container. Aluminum foil was applied to the exterior surfaces of the experimental containers (i.e., the plastic basins and glass tubes) to reduce the effects of light on root growth. The basal medium used in all of the experiments was half-strength Kimura B solution [60]. The length of the seminal roots was evaluated in this study and measured by a ruler.

### RNA extraction, cDNA synthesis, and quantitative PCR (qPCR)

Total RNA was extracted from two different tissues (shoot and root) of 7-day-old rice seedlings subjected to 0 or 150 mM NaCl for 1 day via an RNeasy Plant Mini Kit (Qiagen) according to the manufacturer’s instructions. To avoid contamination by genomic DNA, no more than 6.2 µg of total RNA was treated with Turbo DNA-*free*™ DNase (Ambion) following the manufacturer’s instructions, and 1.3 µg of DNase-treated total RNA was then subjected to cDNA synthesis using the SuperScript™ IV first-strand synthesis system (Invitrogen) according to the manufacturer’s instructions. qPCR was carried out in a CFX Connect Real-Time PCR Detection System (Bio-Rad) using the QuantiNova™ SYBR® Green PCR Kit (Qiagen). The initial amount of template cDNA in each amplification reaction was 4.16 ng. At least five independent biological replicates were performed for each experiment, and *OsACTIN1* (Os03g0718100) was used as an internal control for qPCR normalization. The 2^−ΔΔCT^ method was used to transform threshold cycle values (Ct) into normalized relative abundance values for mRNA. The sequences of the primers used for qPCR are presented in Additional file 1: Table S2.

### Transgene constructs and isolation of transgenic rice

The full-length coding DNA sequences (CDSs) of *OsERF106MZ* and *GFP* were PCR amplified with or without stop codons and cloned into the pGEM-T Easy vector (Promega). These fragments were subcloned into the binary pCAMBIA-1300 vector, in which their expression was driven by a CaMV 35S promoter, to obtain the constructs *35Sp::OsERF106MZ* [OE1] and *35Sp::OsERF106MZ-GFP* [OE2 and OE3]. After the constructs were verified by sequencing, they were transformed into the rice cv. Tainung 67 background for subcellular OsERF106MZ localization and functional assays. Because the stable T_0_ rice transformants were chimeric and to avoid the undesirable side effects of multiple T-DNA insertions, T_1_ seeds were harvested from individual panicles of independent transformant lines that showed a 3:1 ratio of hygromycin resistance and sensitivity normalized to seed viability and were used to further screen homozygous transgenic rice. Additionally, the 2.3-kb *OsERF106MZ* promoter was cloned into pCAMBIA-1305.1 (*OsERF106MZp::GUS*), and the construct was transformed into the rice cv. Tainung 67 background for investigation of the spatial pattern of *OsERF106MZ* expression. The spatial *OsERF106MZ* expression assay was carried out as previously described [61].

### RNA sequencing (RNA-seq) and analysis

For this experiment, the seedlings were transferred to basal medium supplemented with 0 or 150 mM NaCl after growing for 7 days in plastic basins. Twenty-four hours later, the belowground parts of the plants were subjected to RNA extraction according to the method described above. Three biological replicates were performed, and each replicate comprised belowground tissues from more than 5 seedlings. Sequencing libraries were generated with 1.0 µg of RNA input via a KAPA mRNA HyperPrep Kit (Roche) according to the manufacturer’s instructions. Libraries with different indexes were sequenced on a NovaSeq 6000 system (Illumina) according to the manufacturer’s instructions. The original data obtained by high-throughput sequencing were transformed into raw sequence reads by CASAVA base recognition (Base Calling). Raw paired-end sequence reads were filtered and trimmed using Trimmomatic V.0.38 [62] with the following parameters: LEADING:3 TRAILING:3 SLIDINGWINDOW:4:20 MINLEN:36. Clean paired-end reads were aligned to the rice genome (IRGSP-1.0) using HISAT2 V2.1.0 [63]. All the RNA-seq data are available from the Gene Expression Omnibus (GEO, https://www.ncbi.nlm.nih.gov/geo) under accession number GSE213466. To identify DEGs, the RNA-seq data were analyzed with the DEGseq R package [64]. Each up- and downregulated DEG exhibited a log_2_-fold change > 1 and < -1, respectively, with a *P value* < 0. 05 based on the false discovery rate (FDR)-corrected Student’s *t* test. Each DEG is listed and described in the corresponding Supplementary Data.

### ABA ELISA

For this experiment, the seedlings were transferred to basal medium supplemented with 0 or 150 mM NaCl after growing for 7 days in plastic basins. Forty-eight hours later, the belowground parts of the plants were subjected to an immunological ABA assay. Before starting measurements, the samples were vacuum dried at -20 °C overnight to obtain the native dry weight for normalization. The dried samples were ground using a Cell Destroyer PS2000 (Bio Medical. Science Co., Ltd., Tokyo, Japan) before the addition of extraction buffer. The extraction, purification, and quantification of ABA were carried out following the method of Lin et al. [65]. At least five biological replicates, each with two technical replicates, were performed in this experiment.

### Chromatin immunoprecipitation (ChIP)-qPCR assay

Detached belowground tissues from 14-day-old OE3 seedlings were used for ChIP-qPCR assays. Three biological replicates were used, and each replicate comprised belowground tissues from more than 40 seedlings. Each of the three replicate samples was soaked in 30 mL of PBS (137 mM NaCl, 2.7 mM KCl, 10 mM Na_2_HPO_4_, and 1.8 mM KH_2_PO_4_) supplemented with 2 mM disuccinimidyl glutarate (ThermoFisher), and a vacuum was applied three times for 10 min each time. Thereafter, each of these samples was cross-linked in 30 mL of PBS supplemented with 1% (v/v) formaldehyde, and a vacuum was applied three times for 10 min each time. To stop the reaction, 2.0 mL of 2 M glycine was added, and a vacuum was applied for 15 min. Subsequently, chromatin extraction and immunoprecipitation procedures were performed according to the method described by Poza-Viejo et al. [66]. The chromatin complexes were immunoprecipitated using anti-GFP antibody (Abcam, ab290)-coupled magnetic Protein G beads (Cytiva, Code no. 28967066 [IP +]), while protein G beads (IP-) alone were used as a control. The immunoprecipitated DNA was recovered in water and then subjected to ChIP-qPCR assays. The fold enrichment was determined following the method of Haring et al. [67]. The sequences of the primers used for ChIP-qPCR are presented in Additional file 1: Table S2.

### Dual LUC reporter assay

For the dual LUC assay, the 1.0-kb *OsAO3* promoter was amplified by PCR. The PCR product was then cloned into the pGEM-T Easy vector and sequenced. Subsequently, the 1 kb promoter fragment was subcloned into the pJD301 vector [68] to drive the *firefly* LUC gene used as the reporter. The effector plasmid carried the full-length CDS of *OsERF106MZ* under the control of the CaMV 35S promoter. To minimize experimental variability, *Renilla* LUC activity was used as an internal control. To delete the GCC (5’-AGCCGCC-3’) box, the QuikChange Lightning Site-Directed Mutagenesis Kit (Agilent) was used following the manufacturer’s instructions. The reporter plasmid was either transfected alone or cotransfected with the effector plasmid into rice protoplasts isolated from green tissues of 7- to 10-day-old seedlings using PEG-mediated transformation [69]. After 24 h of incubation of the transfected rice protoplasts at 24 °C, LUC activity was assayed with the Dual-Luciferase® Reporter Assay System (Promega). The results are presented as normalized ratios between the activities of the reporter *firefly* LUC and control *Renilla* LUC.

### Statistical analysis

Two-way ANOVA followed by Fisher’s LSD multiple comparison was used to determine significant differences.

## Supplementary Information

**Additional file 1:** **Figure S1.** Molecular identification of OsERF106MZ-overexpressing transgenic rice. a The overexpression of OsERF106MZ in transgenic rice lines was confirmed using q-PCR. b Detection of the OsERF106MZ-GFP fusion protein in transgenic rice lines by Western blotting with anti-GFP antibodies. Upper panel, Coomassie blue staining of total protein as a loading control. Lower panel, Aliquots of 30 µg of protein immunoblotted with anti-GFP antibodies. The images of the bands visualized by Coomassie brilliant blue staining and Western blotting were taken from the same SDS-PAGE gel and PVDF membrane, respectively. **Table S1.** Partial promoter sequences of OsAO3 and OsNPC3 genes. **Table S2.** Primers used in this study.

**Additional file 2.** 

**Additional file 3.** 

**Additional file 4.** 

## Data Availability

All the datasets analyzed in the current study are available in the Gene Expression Omnibus (GEO, https://www.ncbi.nlm.nih.gov/geo) repository under an accession number of GSE213466.

## Abbreviations

ABA: Abscisic acid
AP2/ERF: APETALA2/ethylene-responsive factor
CAT: Catalase
ChIP: Chromatin immunoprecipitation
CRE: *cis*-Regulatory element
DEG: Differentially expressed gene
ET: Ethylene; GFP, green fluorescent protein
GUS: β-Glucuronidase
JA: Jasmonic acid
LR: Lateral root
LUC: Luciferase
MDA: Malondialdehyde
NBT: Nitro blue tetrazolium
NR: Nodal root
PR: Primary root
Qpcr: Quantitative PCR
RNA-seq: RNA sequencing
ROS: Reactive oxygen species
RSA: Root system architecture
SR: Seminal root
TF: Transcription factor
TNG67: Tainung 67 (*Oryza sativa* L. *spp. japonica* cv. Tainung 67)
WT: Wild-type
