# Supplementary material for: Overexpression of OsERF106MZ promotes parental root growth in rice seedlings by relieving the ABA-mediated inhibition of root growth under salinity stress conditions
Source: BMC Plant Biol. 2023 Mar 16;23:144. doi: 10.1186/s12870-023-04136-8 (PMC10018881; doi:10.1186/s12870-023-04136-8)
Supplement: Supplementary file 1 — Additional file 1: Figure S1. Molecular identification of OsERF106MZ-overexpressing transgenic rice. a The overexpression of OsERF106MZ in transgenic rice lines was confirmed using q-PCR. b Detection of the OsERF106MZ-GFP fusion protein in transgenic rice lines by Western blotting with anti-GFP antibodies. Upper panel, Coomassie blue staining of total protein as a loading control. Lower panel, Aliquots of 30 µg of protein immunoblotted with anti-GFP antibodies. The images of the bands visualized by Coomassie brilliant blue staining and Western blotting were taken from the same SDS-PAGE gel and PVDF membrane, respectively. Table S1. Partial promoter sequences of OsAO3 and OsNPC3 genes. Table S2. Primers used in this study. [file 12870_2023_4136_MOESM1_ESM.pdf]

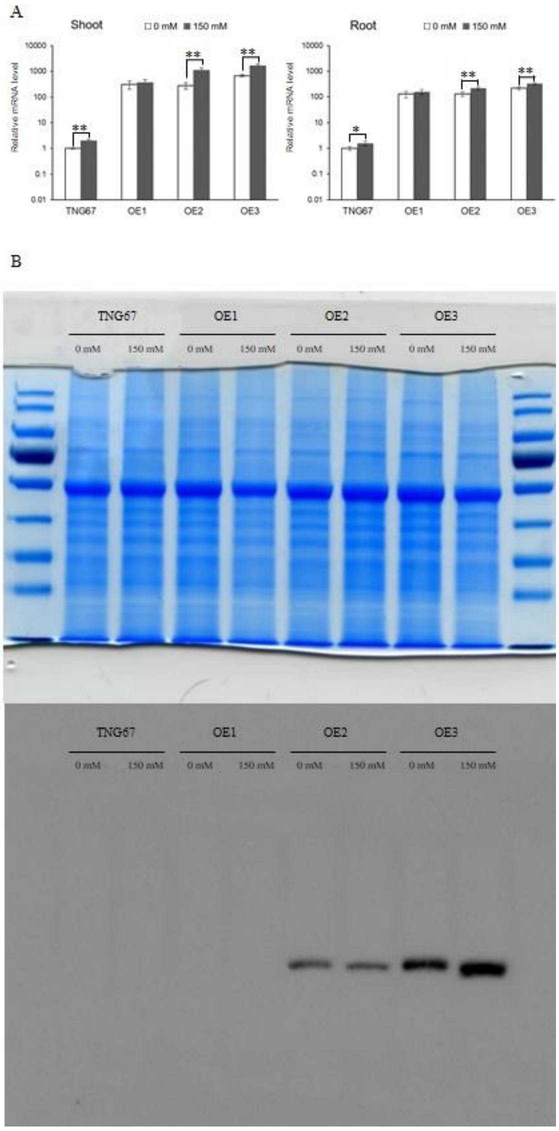

Figure S1. Molecular identification of *OsERF106MZ*-overexpressing transgenic rice. **a** The overexpression of *OsERF106MZ* in transgenic rice lines was confirmed using q-PCR. **b** Detection of the OsERF106MZ-GFP fusion protein in transgenic rice lines by Western blotting with anti-GFP antibodies. Upper panel, Coomassie blue staining of total protein as a loading control. Lower panel, Aliquots of 30 µg of protein immunoblotted with anti-GFP antibodies. The images of the bands visualized by Coomassie brilliant blue staining and Western blotting were taken from the same SDS-PAGE gel and PVDF membrane, respectively.

Biological Process

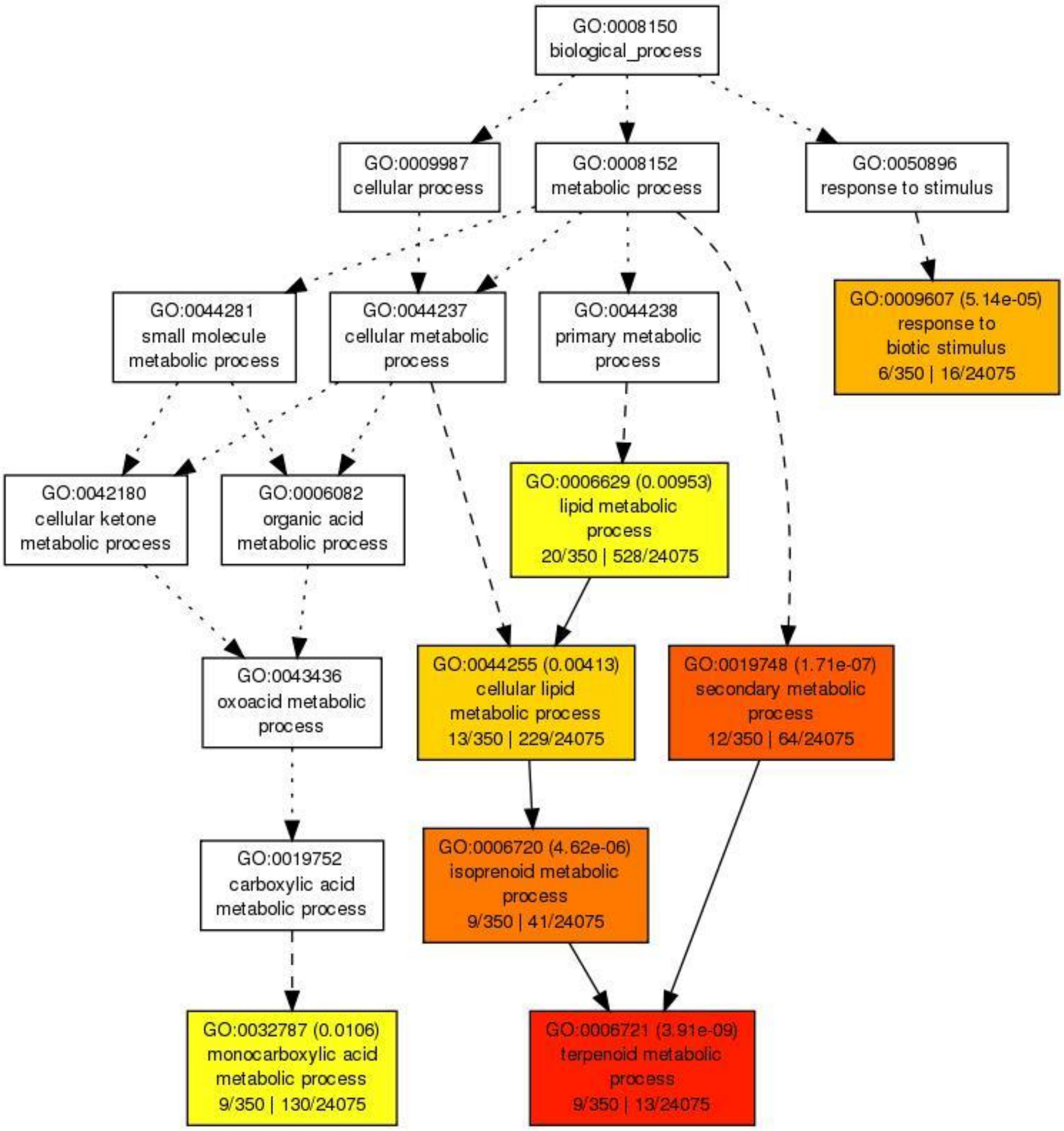

Figure S2. Go analysis of the common DEGs according to biological processes, cellular component, and molecular functions.

continued

Cellular Component

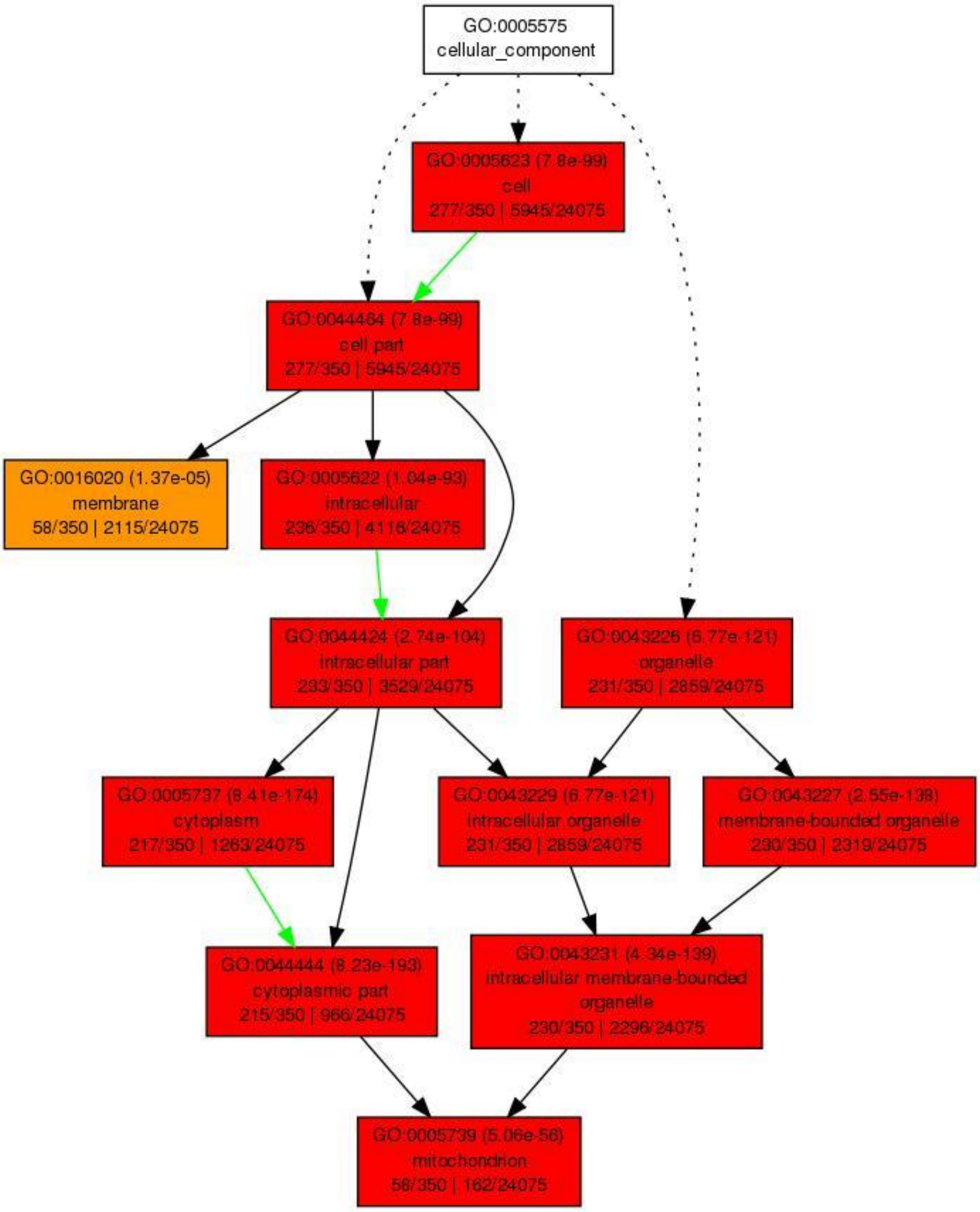

continued

Molecular Function

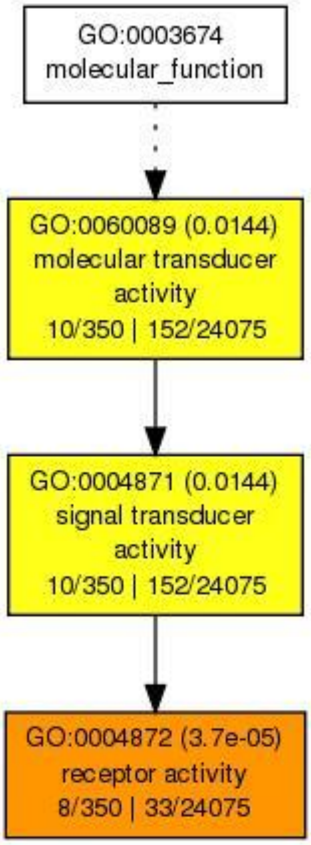

Table S1. Partial promoter sequences of *OsAO3* and *OsNPC3* genes.

GCC BOX: AGCCGCC

>*OsAO3* promoter

CATTATATTAAGGGATGGAGGGAGCAGTGTTTTAATTTTGGTCAAAGGGGATCACCTATACATTTGTCGACAAATTT
TCTCCTAAAAATTTGACAGATATAATTATAGTACAATCGTAGTGTAATTATACTATAACTTGCATGTAACTACAGTGTAA
CTTGTATGTATGTTTTACGTAATTTTGAATAGTTAGATCTATTGCAAGATTTGTTCTTGTGAGGAAGAAAAAAAATCA
CAGCACACATATATGAAAGAATTTGTTCCCGCGGCCTCAATTTTACCGAAATAACATGTTACGGAGAGATTTTTGAA
AGTTACATATAAGTTACATGTAAGTTACAATGTAATTACACTACGATTGTACTATAATTACATCTGTCAAATTTTTGGA
ATAAAATTTGTCAACAAATATATAGCAAAATTGTTATGAAAATATATTTAACTATTTATTTAAGGAAACTAATTTAGTAT
TATAAATATTAGTATATATTTATCTATAAACTTAGTTAAATTTAAAGTAGTTATGACTTTGATTAAAGTCAAAACGACTT
ATAATCTAAAACAGATATATAACAGATATATGGAGTAGGTTATTACACTACTAGAAAAGACATTTTTGTAGGCTGCCA
AAACTCATTTTTGCAGGTGAGAGAGAGGTCCGCCTGCACCACAACGACTGCGAAAATCGATGATTTTCATAGTCG
TGGCAACGGCCTACCTGCGAAAATGGAAGGGACGAAAAAAAAATCGCGCCGGGAGCCGCTGCCGCCGCCCGGC
CTGCGCCGTCGCCGCCGGCCTCCCCCCTCCCTCCTCCGGCCAGATCTGGGAGCGAGGGGGGAGGGGGAGCTGC
CGCAGCCGCTCCACGCCCTCCGCCGCCGCCGCCGGGAGCACACGCCACCGCGCCGCCGGGAGCACACGCCACC
GCGCCGCCTCACCTCCTCCTTCACCGCACGCCGCCGCCGCCGGGAGCACACGCCGCCGGGAGCCGCCGCCGCCT
CCGGCCGGCGGCCGGGGAGATGGGAGGGGAGGGGAGAGGAGGAGAGGTGAGGGGAGGGGAGAGGAGGAA
AGGTGAGGGGACTTGGAAAAAATTTAGGCCCACAAGTCTATTTATATTAAGTCTATTTTCACAGGCGGACCACTTA
AGCGGCGCCTGCGTCCGCCTGCGAAACATTAAATTTCACCCCGATTTATATTTTTATAGACGGTTATTTGGCTCTAA
GAGTCATGTCTGCCTGCGAAAATAAGACTCCTACGTTGGAAAAAATGGTTTTTCAGTGTTATATTTTAAGCCTCACG
CCCATATCCTTGGCCATCGCGCGCGTGTTCGTCGTGCTCGTCGCGCGGAGTAGTGCACACGTCCATCCGTCGGGGT
CGCGGAATGGGACGCGTCCGCCGTGGAGCGGCACTCGCCGTACGTACGTCACGCAACCCAACACATTAGCTCAG
CTAGCTGCCTCTCCATCGCATTTAAACTCCTCCCCTATAGGCAGCGATCGATCGGTCACCCGCCCCCGCGCGCGCGC
GCTGACAAACGCACGCACGCCGCTGGTTGTTGGCAATCGAGCGTGCGAGCGAGAGAGAAACGGAGGAGGAGG
GGACGGCCGGAGAAGACGACGACGACGAGGTGGAGAGATCGGATCATG

>*OsNPC3* promoter

GCCGAACAGTTTAGTACTGGGCTTACCGAGGCCTGCAAAACTTTGGGCCACCAAGGGCCTATAATATTTTTAGGG
AAAAAGTCTATTTAGGCCCTGTACAAGTACATTAGTACTCCGGCCTATTGCCACGGATTATCTAGAACTATATGGATG
AATTGAAGTTTTAAAAACTTAACTTAACAAATAATTTAAAATGAGTGGCTAAATAATACGTGGAAGCATATTTGAATT
ATTGTGTTGTTAAACTAGGCATAGTATAGAGTGTACGTTGAAACAAAAGAGAAAGAAATAAGAGTTGTTGCTGAA
GTTGAAGGTGAAGGTATTCTTAGATCCTCATCAATTTCTATAGGAATATTCCTTTAAAAAATTATTTATTTATTAAGTTT
GAAATAATTAATACACCTACTTAATTAATTACTACCTCCTTCTCTAAATAAAGGATGTTGAACTAACTAGCATCATAAA
AATAAATATGGAGGGAGTATATATTAATGGCACACCTTATTTTACGTGTCTTCCAGTACTTCCTCCGTCTCATAATATA
AGAGATTTTGAATTTTTGCTTGCACTGTTTGACCACTCGTCTTATTCAAAAAATTTATGTAAATGTAAAAAACGAAA
AGTTGTGCTTAAAATACTTTAGATAATAAAGTAAGTAAAAAAATAAATAATAATTTTAAAAATATTTTAAATAAGACGA

GTGTTCAAACAATACAAACAAAAATTCAAAATTCTTTATATTATGGGACGGAGGGAGTATTCTTCTTTCCTCTACTTT

CAAACATAAACATGAGAAGAGTACGCTGTACATAATAACCTAATACAGTAATTTTTTTTAATGAACATAACAACCTAC

TACGACGTACTAATAATTAATCATGTCACCTCAGCAATTGCAAGGATTTGCTTAATTAACAAAATGAGACTACTTGTA

CATACTGTATTTAGCAGCATCTCACACGTCTCCACCACACGAG**AGCCGCC**AATAAATACACATAGAAGCAGCTACTA

TCAGCTCGCCACTGAGCTCTAGCTAATAAGCCATCCTCGATCGTTTATCTTCCTCTTCCCTCTTCCAATCCCAGCCTC

GACGCCAGCC**ATG**

Table S2. Primers used in this study.

PCR Type	Gene	Sequence (5’-3’)	Annotation
qPCR	*OsERF106MZ*<br>Os08g0537900	GGTTTCTGCCGGACTAACGA<br>GATGGTCCAAAGGGAGGCTT	108 bp
	OsAO3<br>Os07g0281700	ACAACTGGGGTGCTCTTTCA<br>AGATTCTCCTGCGCTGTCTG	224 bp
	OsNPC3<br>Os11g0593000	GCTCTTCTACAGGAACATGAGG<br>GCTCGATGACGACGTAGTTT	116 bp
	OsMYB91<br>Os12g0572000	TCGAGCGCTGGAAGAACTACCT<br>GCGATCTTCTTCCACTTGTTGC	118 bp
	OsSPX-MFS1<br>Os04g0573000	GCGATTCTTGGGTGTACTGT<br>CGTGAAAGCAACGACAGGTT	233 bp
	OsWRKY76<br>Os09g0417600	TTCCGAATGCTTTTCTGCTG<br>ATCGTGAGGCCCGATAGAAG	111 bp
	OsACTIN1<br>Os03g0718100	CTCAGCACATTCCAGCAGATGTG<br>GATAACAGCTCCTCTTGGCTTAGC	126 bp

PCR Type	Amplicon	Sequence (5’-3’)	Annotation
ChIP-qPCR	*AO3-a*	GGGGATCACCTATACATTTG<br>GTTACACTGTAGTTACATGC	112 bp
	AO3-b	TCACCTCCTCCTTCACCGCACG<br>GCTTAAGTGGTCCGCCTGTG	214 bp
	AO3-c	GCTGGTTGTTGGCAATCGAG<br>CTCGTCGTCGTCGTCTTCTC	80 bp
	NPC3-a	CAGTTTAGTACTGGGCTTAC<br>AATAGGCCGGAGTACTAATG	118 bp
	NPC3-b	TTAGCAGCATCTCACACGTC<br>GGGATTGGAAGAGGGAAGAG	139 bp
	NPC3-c	TCAGTAGCATGGCAGCTAGC<br>TGTCTTCATGTGCGGTTTGG	147 bp
